# Molecular identification of the chitinase genes in *Aedes albopictus* and essential roles of *AaCht*10 in pupal-adult transition

**DOI:** 10.1186/s13071-023-05733-0

**Published:** 2023-04-01

**Authors:** Sha An, Wenjuan Liu, Jingwen Fu, Zhong Zhang, Ruiling Zhang

**Affiliations:** 1 Shandong Provincial Hospital Affiliated to Shandong First Medical University, Jinan, 250000 China; 2 School of Clinical and Basic Medical Science, Shandong First Medical University (Shandong Academy of Medical Sciences), Jinan, 250117 China; 3 School of Laboratory Animal (Shandong Laboratory Animal Center), Shandong First Medical University (Shandong Academy of Medical Sciences), Jinan, 250117 China

**Keywords:** Asian tiger mosquito, Chitin, Mosquito control

## Abstract

**Background:**

*Aedes albopictus* is an increasingly serious threat in public health due to it is vector of multiple arboviruses that cause devastating human diseases, as well as its widening distribution in recent years. Insecticide resistance is a serious problem worldwide that limits the efficacy of chemical control strategies against *Ae. albopictus*. Chitinase genes have been widely recognized as attractive targets for the development of effective and environmentally safe insect management measures.

**Methods:**

Chitinase genes of *Ae*. *albopictus* were identified and characterized on the basis of bioinformatics search of the referenced genome. Gene characterizations and phylogenetic relationships of chitinase genes were investigated, and spatio-temporal expression pattern of each chitinase gene was evaluated using qRT-PCR. RNA interference (RNAi) was used to suppress the expression of *AaCht*10, and the roles of *AaCht*10 were verified based on phynotype observations, chitin content analysis and hematoxylin and eosin (H&E) stain of epidermis and midgut.

**Results:**

Altogether, 14 chitinase-related genes (12 chitinase genes and 2 *IDGF*s) encoding 17 proteins were identified. Phylogenetic analysis showed that all these *AaCht*s were classified into seven groups, and most of them were gathered into group IX. Only *AaCht*5-1, *AaCht*10 and *AaCht*18 contained both catalytic and chitin-binding domains. Different *AaCht*s displayed development- and tissue-specific expression profiling. Suppression of the expression of *AaCht*10 resulted in abnormal molting, increased mortality, decreased chitin content and thinning epicuticle, procuticle and midgut wall of pupa.

**Conclusions:**

Findings of the present study will aid in determining the biological functions of *AaCht*s and also contribute to using *AaCht*s as potential target for mosquito management.

**Graphical Abstract:**

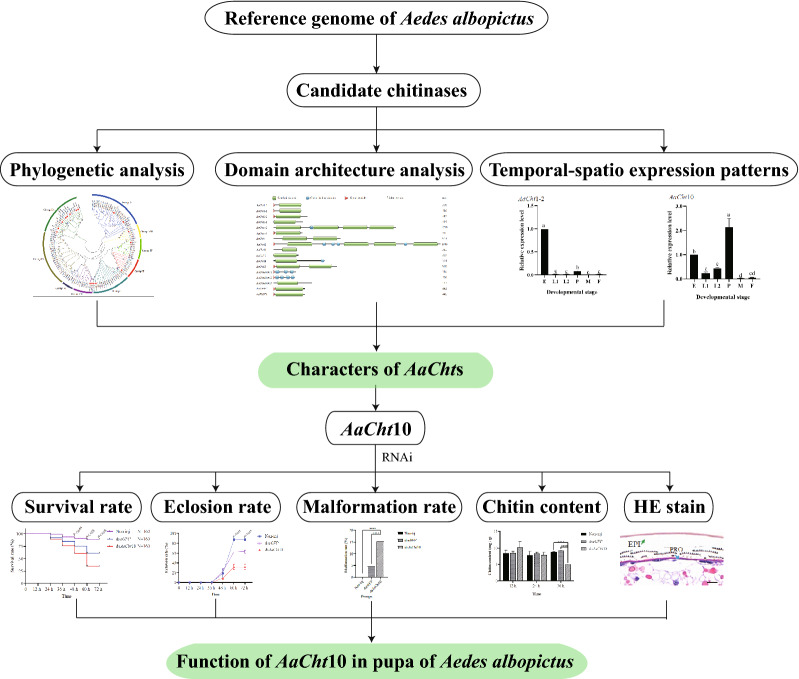

**Supplementary Information:**

The online version contains supplementary material available at 10.1186/s13071-023-05733-0.

## Background

Arthropod-borne viruses transmitted by *Aedes* mosquitoes, such as dengue, yellow fever, Zika and chikungunya viruses, have been expanding their global distribution in recent years, causing significant human morbidity and mortality in affected regions [1–4]. No specific therapeutic treatment or effective vaccine is available for these arboviruses, and vector control remains the primary public health intervention to prevent and respond to epidemics [5]. As one of the major vectors of these arboviruses, *Aedes albopictus*, originating in Southeast Asia, has invaded more than 70 countries and regions worldwide [1]. Further global expansion is expected concerning the increases in global temperature, urbanization and travels [6, 7]. Chemical insecticide is widely used in mosquito-borne disease control and prevention [8, 9]. Besides environmental pollution and non-specific toxicity, frequent and unprecedented quantity of insecticide use exerts an exceptionally strong selective pressure for resistance. Insecticide resistance of mosquitoes to most of the WHO-approved public health insecticides has been reported worldwide [10–12].

Chitin is a polymer of β (1,4)-linked N-acetylglucosamine, which is the critical constituent of arthropod exoskeletons (cuticle) and the peritrophic membrane (PM) in midgut [13, 14]. The tough and durable exoskeleton provides physical support and protects insects from external damage, which also restricts the growth of insects [15]. Therefore, the old chitin degraded and new chitin is synthesized periodically to allow for molting and metamorphosis of insects [16]. As chitin is an indispensable structure for survival of insects, the balance of chitin content is crucial for individual development. Meanwhile, chitin is absent in animals and plants [17]. Hence, chitin could be used as target for mosquito control; altering the metabolism pathways to intervene formation or degradation of chitin would be helpful for exploiting novel and environmentally friendly biological control strategies.

Chitinases (Chts) are one of the largest groups of hydrolases that break down glycosidic bonds in chitin and decompose chitin into N-acetylglucosamines [18]. Several studies have been explored utilizing chitinase genes as biocontrol molecule agent to interrupt the molting process of insects, such as *Ostrinia nubilalis* (Lepidopteran) [19], *Tribolium castaneum* (Coleopteran) [20], *Anopheles gambiae* (Dipteran) [21] and *Locusta migratoria* (Neopteran) [22], resulting in abnormal molting and increased mortality. Based on conserved amino acids, protein folding and conserved motifs, chitinases are classified into glycosyl hydrolase family 18 (GH18) and family 19 (GH19) [23]. These two chitinase families possess distinct sequence features and three dimensional (3D) structures; all insect chitinases belong to the GH18 [24]. Besides chitinases, some Cht-like proteins that lack chitinase activity, such as imaginal disc growth factors (*IDGF*s), are also included in GH18 [24]. Previous studiees have demonstrated that the number of chitinase genes varies in different species; chitinases and Cht-like proteins can be classified into different groups according to amino acid similarities and phylogenetic relationships, and functions of chitinases differ greatly among groups [15, 25]. Several chitinases were found to be essential for insect survival, molting and development [26–28]. Downregulating the expression level of insect chitinase genes results in severe phenotypes, including ecdysis disturbance, growth inhibition, pupation failure and death [29].

To explore members of chitinase in *Ae*. *albopictus* and the potential to use chitinases as promising targets for mosquito control, a systematic genome-wide investigation of *Ae*. *albopictus* chitinase genes was performed. The developmental- and tissue-specific expression patterns of all chitinases in *Ae*. *albopictus* (*AaCht*) were profiled to identify optimal candidate genes that can be used as a target to disrupt the chitin metabolism pathway. Previous work has demonstrated that Group II chitinase (ChtII, also known as chitinase 10) has multiple catalytic and chitin-binding domains, which are indispensable for insect ecdysis at all developmental stages [30]. The functions of chitinase 10 (Cht10) have been assessed by RNA interference (RNAi), and molting defects have been detected in many insect species [25, 31–33]. Considering the importance of Cht10 in insects, the function of *AaCht*10 was explored using RNAi to provide insights to design mosquito control strategy utilizing chitinase.

## Materials and methods

### Mosquito maintenance

*Aedes albopictus* samples used in this study were from a colony collected in Shandong Province (China) maintained in a laboratory and reared at 27 ± 1 °C and 65% relative humidity (RH) with a daily photoperiod of 14:10 h (L:D). Adults were maintained in a 10% sucrose solution, and the females were fed mouse blood for egg-laying. The larvae were reared on slurry that was a mixture of pork liver powder (homemade), yeast and distilled water.

### Chitinase gene identification and phylogenetic analysis

Chitinase genes of *Aedes aegypti* (annotated based on Genome version: GCA_000004015.3) and *Culex quinquefasciatus* (annotated based on Genome version: GCA_015732765.1) downloaded from VectorBase database (https://www.vectorbase.org) were used as query to screen for putative chitinases and *IDGF*s genes in the reference genome of *Ae*. *albopictus* (Genome version: *Aalb*F2, assembly: GCA_006496715.1, NCBI) [34]. Default parameters were used for the analyses (*E*-value cutoff = 1.0e− 5). Identified candidate chitinase and *IDGF* genes were compared with the protein database of *Ae*. *albopictus* in VectorBase using the BLASTp program [35] with the default settings.

Molecular weight, amino acid numbers and theoretical isoelectric points (pIs) of the chitinase sequences were calculated using ExPASy Proteomics Server (http://cn.expasy.org/tools/pitool.html) [36]. SMART (http://smart.emblheidelberg.de/) was used to confirm the chitin binding, catalytic and transmembrane domains. Multiple sequence alignment and identification of conserved domains were performed using MAFFT (http://mafft.cbrc.jp/alignment/server/). Then, the graphical displays of chitinase gene sequences were created using the online Gene Structure Display Server (2.071) (http://gsds.cbi.pku.edu.cn/).

Altogether, 123 sequences from nine species (*Ae. albopictus*, *Aedes aegypti*, *An. gambiae*, *Bactrocera dorsalis*, *Cx. quinquefasciatus*, *Drosophila melanogaster*, *Nilaparvata lugens*, *T. castaneum* and *Plutella xylostella*) (Additional file 1: Table S1) were used to explore the evolutionary relationships of chitinases. The ClustalW alignment function in MEGA 7.0 [37] was used to align all chitinase sequences. An unrooted neighbor-joining (NJ) phylogenetic tree was constructed using MEGA 7.0 with 1000 bootstrap replicates. Chitinase and *IDGF* genes in this study were named as *AaCht* and *AaIDGF*; a homology sequence of each gene was represented with numbers.

### Total RNA extraction and cDNA synthesis

To evaluate the expression pattern of chitinase genes, samples of different developmental stages and various tissues of pupa were prepared, respectively. Two hundred eggs were collected within 24 h after deposition by blood-fed females, and they were pooled to represent the embryonic stage. Larvae samples were divided into early (I–II instars) and late (III–IV instars) larval stage; 100 early larval and 50 late larval were collected, respectively. Fifty pupae at 12–24 h after pupate were mixed. Fifty male and fifty female adults were collected separately within 12 h after eclosion.

Pupae were first immobilized on an ice box for 6 min and then transferred to precooled PBS solution for dissection, which was performed with forceps while using a dissecting microscope. Four different tissues (cephalothorax, integument, midgut and malpighian tube) from 100 pupae were collected, respectively. All samples were flash frozen in liquid nitrogen immediately following collection and then stored at − 80 °C until RNA isolation.

Total RNA was extracted using RNA isolater total RNA extraction reagent (Vazyme, China) and treated with DNase I (Vazyme, China) to remove genomic DNA. cDNA was synthesized from 1 μl total RNA using HiScript 3 RT SuperMix for qPCR (Vazyme, China) according to the manufacturer’s instructions. The quality and quantity were detected by 2% agarose gels and ScanDrop spectrophotometer (Jena, Germany).

Real-time quantitative PCR (qRT‒PCR) was carried out using ChamQ Universal SYBR qPCR Master Mix (Vazyme, China) on an ABI7500 qRT-PCR platform (Thermo Fisher Scientific, USA). Primers used for qRT-PCR were listed in Additional file 1: Table S2. All qRT-PCRs were carried out with 20 μl reaction mixture consisting of 10 μl qPCR Master Mix, 2 μl cDNA templates and 0.4 μl each of forward and reverse primers. The processes were 95 °C for 30 s, 40 cycles of 95 °C for 10 s and 60 °C for 30 s. Melting curve analysis from 65 °C to 95 °C was conducted to verify a single PCR product. The expression levels of chitinase genes were normalized against *β*-actin. All experiments were performed in triplicate and repeated three times. The 2^−ΔΔCT^ method was used to estimate the relative expression of chitinase gene [38]. *P* value < 0.05 was considered statistically significant.

### Molecular cloning of AaCht10 and double-stranded RNA synthesis

The open reading frame (ORF) of *AaCht*10 was predicted using the ORF Finder (http://www.ncbi.nlm.nih.gov/gorf/gorf.html), and E-RNAi (http://www.dkfz.de/signaling/e-rnai3/idseq.php) was used to confirm target sequence of *AaCht*10 and enhanced green fluorescent protein (*eGFP*, used as control) (GenBank accession number: CAA58789). Then, the forward and reverse primers harboring T7 RNA polymerase promoter were designed according to the target sequence of *AaCht*10 and *eGFP*. Polymerase chain reaction (PCR) was used to amplify the target sequence of *AaCht*10 using 2 × Phanta Max Master Mix Kit (Vazyme, China) with specific primers (Additional file 1: Table S3). PCR reactions were set up in total volume of 50 μl, consisting of 25 μl Phanta Max Master Mix, 5 μl template DNA and 2 μl each of forward and reverse primers. PCR was performed by initially denaturing the cDNA template for 3 min at 95 °C followed by 35 cycles consisting of 15 s at 95 °C, 15 s at 60 °C, 60 s at 72 and a final extension step for 5 min at 72 °C. FastPure Gel DNA Extractin Mini Kit (Vazyme, China) was used to purify the PCR product, which was then cloned into a 5 min TM TA/Blunt-Zero Cloning Kit (Vazyme, China) for sequencing from both directions. The positive recombinant plasmid was purified using FastPure Plasmid Mini Kit (Vazyme, China). Afterwards, using the same PCR conditions as mentioned above, the plasmid DNA was amplified and used as template for synthesis of ds*AaCht*10 and ds*eGFP* using T7 RNAi Transcription Kit (Vazyme, China).

### Verification of RNAi-mediated AaCht10 silencing

The concentration of purified dsRNA was measured using ScanDrop spectrophotometer (Jena, Germany). The pupae at 12 h after pupate were selected for injection. Microinjector was carried out using Nanoject III (Drummond, USA); 750 ng (0.025 μl of 0.3 μg/μl) ds*AaCht*10 was injected into pupa from the dorsal cuticle between the thorax and abdomen under the dissecting microscope [39]. Two control groups, i.e. ds*eGFP* group (pupae injected with ds*eGFP*) and control (non-injected pupae, Non-inj) were used in this study. All experiments were repeated three times.

Living pupae were collected at 12 h, 24 h and 36 h after dsRNA treatment, respectively. qRT-PCR was performed to evaluate the effects of ds*AaCht*10 on gene expression. The survival, malformation and eclosion rates were analyzed at 12 h, 24 h, 36 h, 48 h and 72 h after ds*AaCht*10 injection had been performed. Malformation phenotypes were observed under optical microscope, and the Leica Application Suite V4 program was used to take photos (Leica Microsystems, Switzerland).

### Analysis of chitin content after RNAi

The method used to estimate chitin content referred to Arakane et al. (2005) [40] with some modifications. To be specific, samples at 12 h, 24 h and 36 h after treatment were collected and put into an oven (65 °C for 1 h). Then, dried samples were weighed and added to a glass grinding tube containing 1 ml sterilized ddH_2_O. Homogenates were transferred to new microfuge tubes (1.5 ml) and centrifuged at 5000 r/min for 15 min at room temperature, and the supernatant was discarded. The pellet was suspended in 400 μl 3% sodium dodecyl sulfate (SDS) and then heated at 100 °C for 15 min. After cooling, samples were centrifuged at 5000 r/min for 10 min, and the supernatant was discarded. The pellet was suspended in 500 μl sterilized ddH_2_O and then centrifuged at 5000 r/min for 10 min, and the supernatant was discarded. We added 300 μl 120% KOH to suspend the pellet and heated the samples at 130 °C for 60 min. Samples were mixed with 800 μl precooled 75% ethanol and put on ice for 15 min. After adding 30 μl 5% diatomite suspension, the samples were stirred and then centrifuged at 5000 r/min for 5 min at 4 °C, and the supernatant was discarded. The pellet was washed once using precooled 40% ethanol and then washed twice using sterilized ddH_2_O. Precooled sterilized ddH_2_O (500 μl) was used to suspend the pellet and then transfer 100 μl suspension to new microfuge tubes (1.5 ml). After mixing with 50 μl 10% NaNO_2_ and 50 μl 10% KHSO_4_, the sample was centrifuged at 5000 r/min for 15 min at 4 °C. Then, the supernatant (60 μl) was transferred to new microfuge tubes and mixed with 20 μl NH_4_SO_3_NH_2_. We added 20 µl freshly prepared 0.5% 3-methyl-2-benzothiazolinone hydrazine (MBTH) to the samples, heated them at 99 °C for 3 min and added 20 μl FeCl_3_ after the samples had cooled down. We transferred 100 μl of each sample to an ELISA plate, and the absorbance at 630 nm was recorded. Using acetylglucosamine (GlcNAc) as template, a standard curve was drawn based on the absorbance of different concentrations (500, 400, 300, 200, 100, 80, 60, 40, 20 and 0 μg/ml).

### Hematoxylin and eosin stain

To explore the effect of *AaCht*10 on chitin metabolism, tissue sections and hematoxylin and eosin (HE) stain were performed for microscopic examination. Pupae at 36 h after injection of ds*AaCht*10 were chosen. Epidermis and midgut sections were obtained by transecting the middle of the third abdomen of the pupae. The dissected samples were fixed using 4% paraformaldehyde and then rinsed with running ddH_2_O for 20 min to remove residual paraformaldehyde. Different concentrations of ethanol were used for dehydration (75% for 3 h, 85% for 1 h, 95% for 1 h and 20 min, 100% for 20 min). Transparency was achieved by soaking samples into ethanol/xylene (1:1) mixture solution for 20 min, 100% xylene for 15 min and 100% xylene for 15 min successively. Embedding in paraffin was performed by soaking samples in melted 100% paraffin for 1 h, newly melting 100% paraffin for 2 h, newly melting 100% paraffin for 3 h and then putting samples into an embedded frame for cooling and solidification. Next, 4-μm paraffin sections were made with a RM2125 RST rotary microtome (Leica, Germany). The sections were adhered to slides, dried for 20 min and then deparaffinized using 100% xylene twice, 10 min each time. After washing using different concentrations of ethanol (100%, 95%, 85% and 75%) and ddH_2_O, the cleaned slide were dyed with hematine for 10 min and then washed use rinsing water for 2 min. Slides were placed in 1% hydrochloric acid (dissolved in ethanol and ddH_2_O) for 10 s and washed with rinsing water for 2 min. Slides were transferred to 50 °C water for 30 s and washed with rinsing water for 2 min. Counterstaining of slides was carried out with eosin for 5 min, and they were washed with rinsing water for 5 s. Finally, slides were covered and images collected using Pannoramic 250 (3DHISTECH, Hungary).

### Statistical analysis

Differences in gene expression levels were assessed using one-way analysis of variance; multiple comparisons were performed post hoc using the Tukey-Kramer honestly significant difference test with Prism 8.0 (GraphPad Software). *P*-value < 0.05 denoted statistical significance. The survival rate was evaluated using the log-rank test and Mantel-Cox test in Kaplan-Meier method.

## Results

### Identification and phylogenetic analysis of chitinase genes in *Ae. albopictus*

Altogether, 14 chitinase-related genes (12 chitinase genes and 2 *IDGF*s) encoding 17 proteins were identified from the genome sequence of *Ae. albopictus* (Table 1). These identified putative chitinase-like genes were assigned gene numbers according to the previously identified members of the other insect chitinase-like gene family to which they are most closely related, while genes lacking homogeny were represented by *Cht*-New (Table 1). The length of predicted chitinase proteins ranged from 306 amino acids (aa) (*AaCht*-New 2) to 2366 aa (*AaCht*10). The relative molecular mass ranged from 34.10 kD (*AaCht*-New 2) to 267.77 kD (*AaCht*10), and the pIs ranged from 4.18 (*AaCht*-New 2) to 8.28 (*AaIDGF*2) (Table 1).

**Table 1:** Information on chitinase genes in* Aedes albopictus*

Gene symbol	Transcript ID in Vectorbase	Length of amino acid (aa)	Molecular weight (kD)	Isoelectric point
*AaCht*1-1	AALF024608-RA	396	44.61	7.27
*AaCht*1-2	AALF004020-RA	396	44.65	7.52
*AaCht*2-1	AALF021117-RA	482	54.18	5.37
*AaCht*2-2	AALF015610-RA	415	46.48	5.22
*AaCht*5-1	AALF023420-RA	1740	195.65	5.39
*AaCht*5-2	AALF008220-RA	411	15.68	5.60
*AaCht*7	AALF024829-RA	954	107.44	7.55
*AaCht*10	AALF010019-RA	2366	267.77	6.98
*AaCht*11	AALF015878-RA	331	37.90	6.90
*AaCht*17	AALF011996-RA	355	38.77	6.34
*AaCht*18	AALF023915-RA	724	79.56	6.42
*AaCht*20	AALF002858-RA	900	99.52	5.20
*AaCht-*New 1	AALF019402-RA	316	34.57	5.32
*AaCht-*New 2	AALF006375-RA	306	34.10	4.18
*AaCht-*New 3	AALF014503-RA	547	61.65	4.78
*AaIDGF*2	AALF002418-RA	442	48.19	8.28
*AaIDGF*4	AALF002417-RA	441	48.71	6.65

**Fig. 1:**
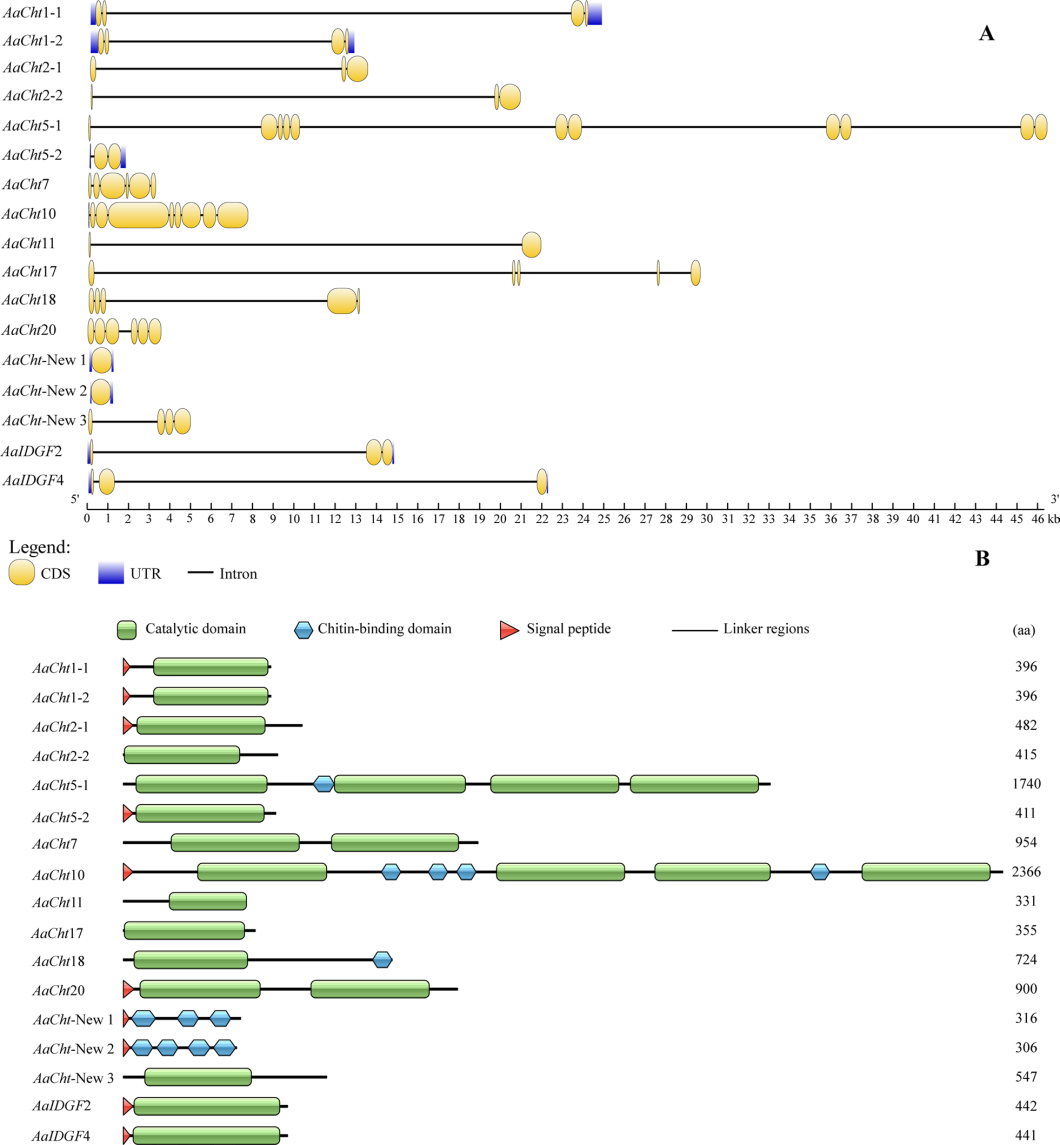
Structural features of *Aedes albopictus* chitinases genes. **A** Intron and exon structure. Yellow, coding sequence; blue, untranslated regions; black lines, intron. **B** Domain architectures. Green rectangle, catalytic domain; blue hexagon, chitin-binding domain; red triangle, transmembrane region; horizontal line, linker regions

All chitinase genes contained at least one exon, and *AaCht*5-1 contained 11 exons, the most of all the chitinase genes (Fig. 1A). Domain architecture analysis demonstrated that a signal peptide was found in 11 chitinase proteins. Except for *AaCht*-New 1 and *AaCht*-New 2, all other chitinase proteins contained the GH18 catalytic domain; four catalytic domains were found in *AaCht*5-1 and *AaCht*10, and two catalytic domains were found in *AaCht*7 and *AaCht*20, respectively. In addition, among all these 17 chitinase proteins, chitin binding domains were only detected in five *AaCht*s; there were 1, 4, 1, 3 and 4 chitin binding domains in *AaCht*5-1, *AaCht*10, *AaCht*18, *AaCht*-New 1 and *AaCht*-New 2, respectively (Fig. 1B).

**Fig. 2:**
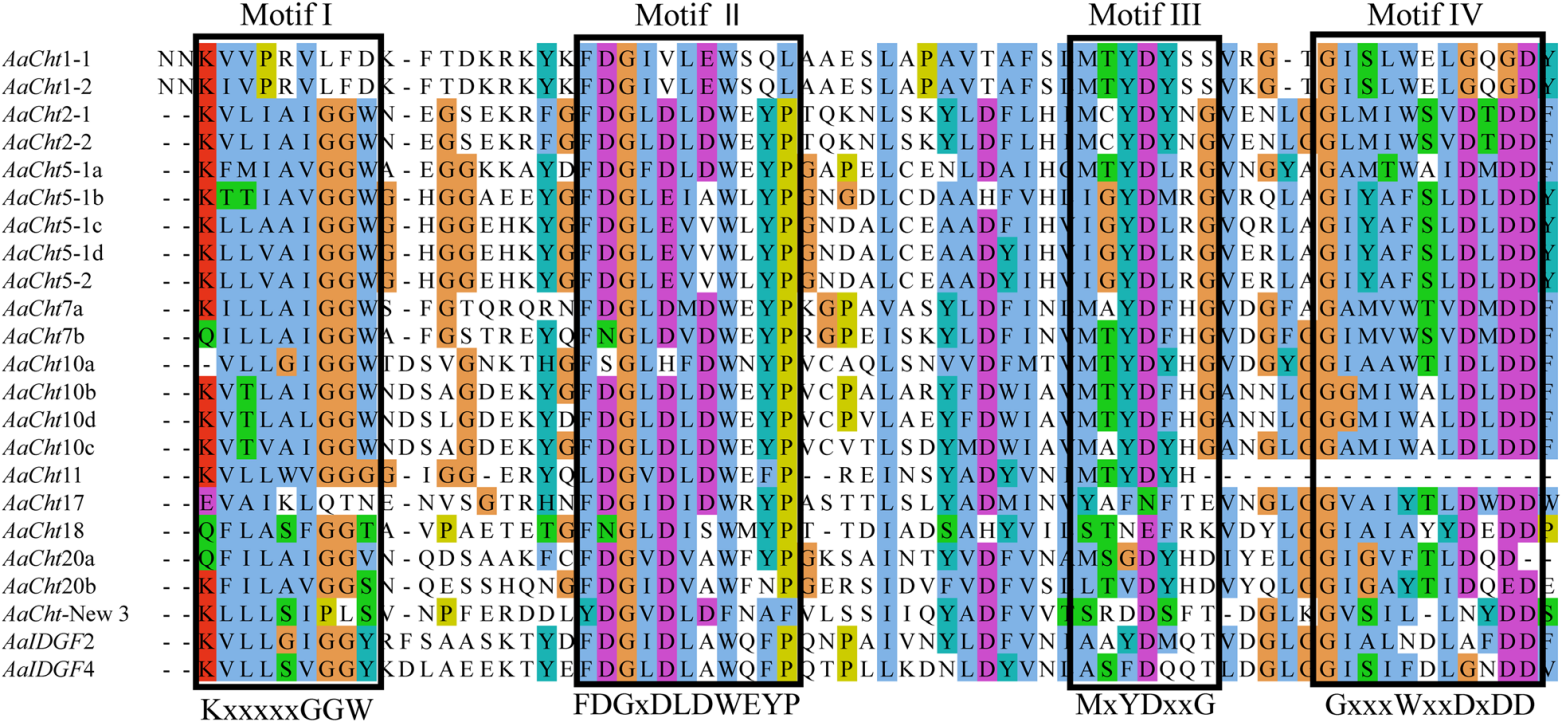
Amino acid sequence analysis of catalytic domain of *Aedes albopictus* chitinases. Four conservative motifs are displayed using black boxes

**Fig. 3:**
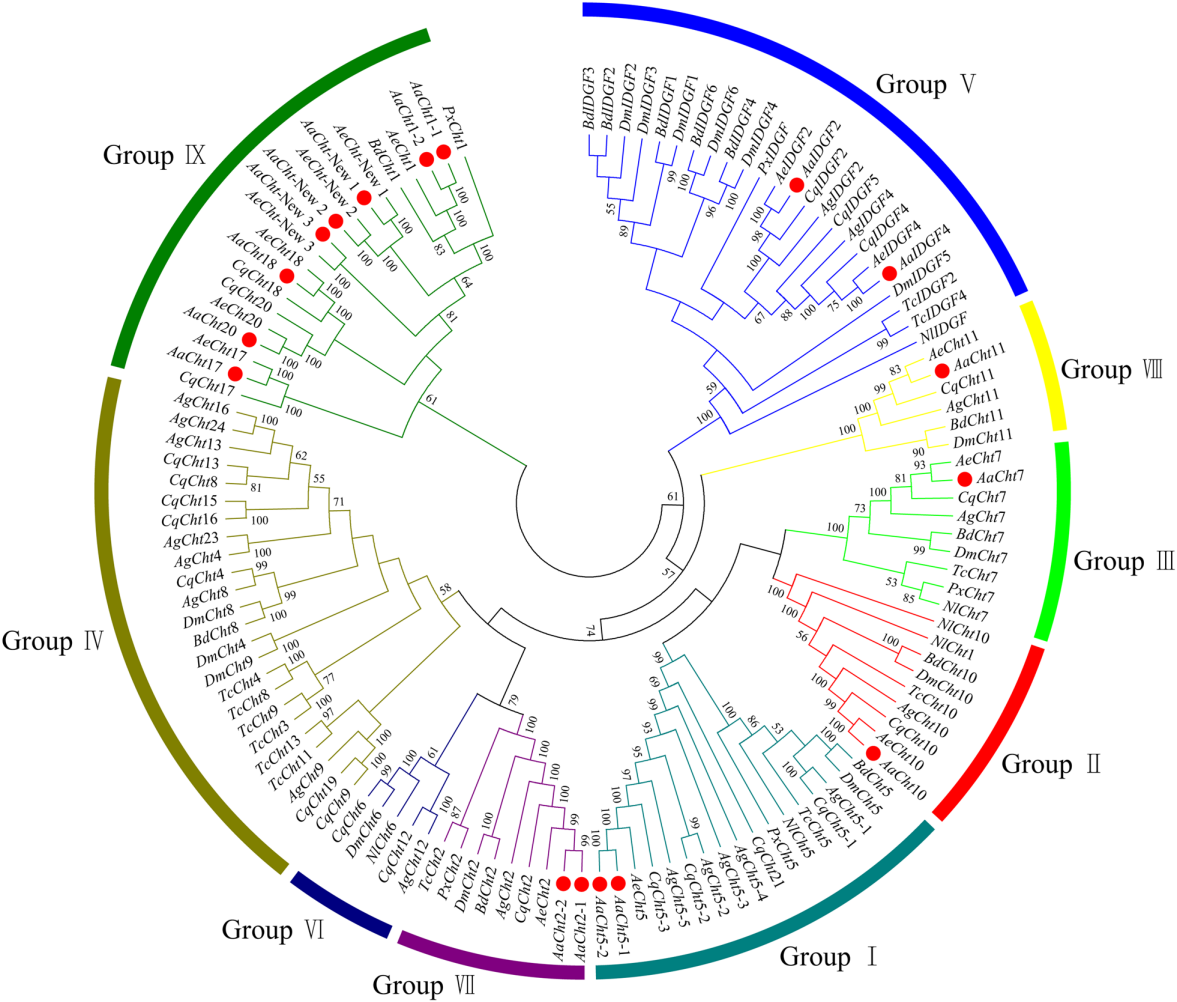
Phylogenetic relationships of chitinases from different species. *Aa*, *Aedes albopictus*; *Ae*, *Aedes aegypti*; *Ag*, *Anopheles gambiae*; *Bd*, *Bactrocera dorsalis*; *Cq*, *Culex quinquefasciatus*; *Dm*, *Drosophila melanogaster*; *Nl*, *Nilaparvata lugens*; *Px*, *Plutella xylostella*; *Tc*, *Tribolium castaneum*. Numbers at branches are bootstrap support values. The red dots represent the chitinases of *Ae. albopictus*

The results of multiple sequence alignments suggested that 16 *AaCht*s have four conserved motifs, KxxxxxGGW (motif I), FDGxDLDWEYP (motif II), MxYDxxG (motif III) and GxxxWxxDxDD (motif IV), whereas *AaCht*11 lacks motif IV (Fig. 2). The constitution of amino acid residue analysis showed that residue E in motif II was retained in *AaCht*2-1, *AaCht*2-2, *AaCht*5-1a, *AaCht*7 (a, b), *AaCht*10 (b, c, d) and *AaCht*11 (Fig. 2). Phylogenetic analysis using 123 amino acid sequences from nine insect species (Additional file 1: Table S1) demonstrated that all these chitinase proteins were clustered into nine distinct groups (I–IX). The 17 chitinase proteins of *Ae*. *albopictus* were divided into seven groups, and most of them were gathered in group IX. Both *AaIDGF*2 and *AaIDGF*4 were clustered into group V with *IDGF* sequences from other species, and only one *AaCht* was included in group II (*AaCht*10), III (*AaCht*7) and VIII (*AaCht*11). Furthermore, there were two *AaChts* in group I (*AaCht*5-1, *AaCht*5-2) and group VII (*AaCht*2-1, *AaCht*2-2), respectively (Fig. 3).

### Temporal-spatio expression patterns of *AaChts*

**Fig. 4:**
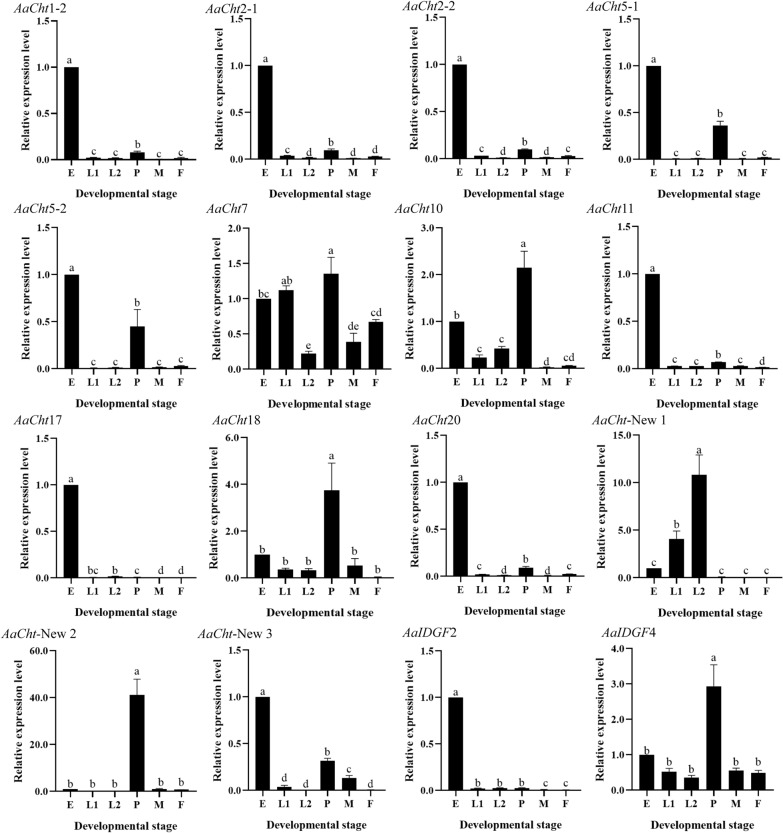
Expression patterns of *AaCht*s in different development stages of *Aedes albopictus*. E, egg; L1, early larva; L2, late larva; P, pupa; M, male; F, female. All data are represented as means ± SE. Different lower case letters (a–d) on the bars indicate significant differences among different samples

The expression level of all *AaCht*s in different developmental stages were evaluated. According to the results of qRT-PCR, *AaCht*17 and *AaIDGF*2 showed high expression merely in eggs; eight *AaCht*s (*AaCht*1-2, *AaCht*2-1, *AaCht*2-2, *AaCht*5-1, *AaCht*5-2, *AaCht*11, *AaCht*20 and *AaCht*-New 3) were highly expressed in egg, followed by pupa; *AaCht*10, *AaCht*18, *AaCht*-New 2, *AaCht*7 and *AaIDGF*4 had peak expression in pupa; *AaCht*-New 1 was expressed at a high level in late larval stages (Fig. 4).

**Fig. 5:**
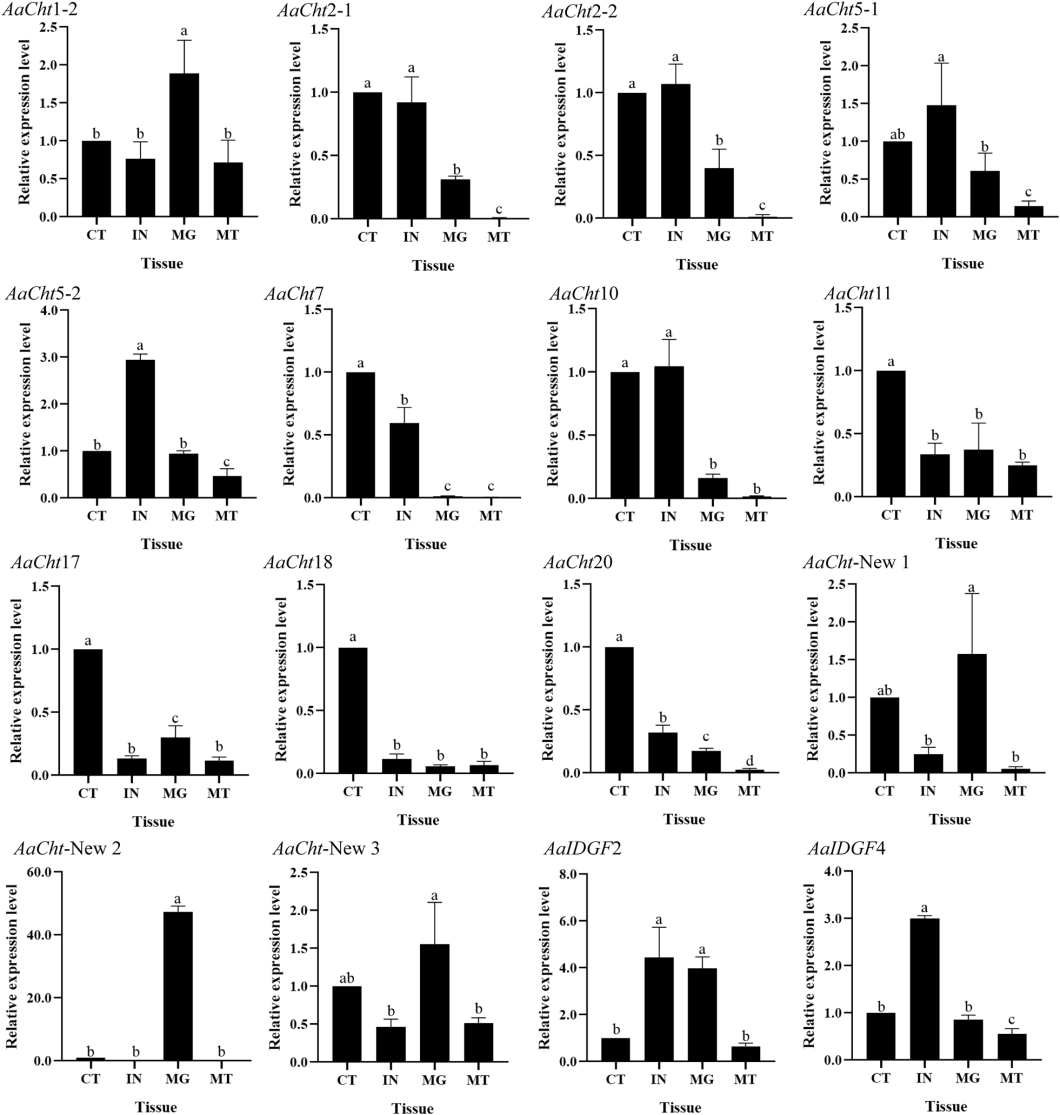
Expression patterns of *AaCht*s in different tissues of *Aedes albopictus* pupae. CT, cephalothorax; IN, integument; MG, midgut; MT, malpighian tube. All data are represented as means ± SE. Different lower case letters (a–d) on the bars indicate significant differences among different samples

Among four tissues of pupae, relatively high expression of *AaCht*2-1, *AaCht*2-2 and *AaCht*10 was found in both cephalothorax and integument; *AaCht*7, *AaCht*11, *AaCht*17, *AaCht*18 and *AaCht*20 were expressed at high levels in cephalothorax; the highest expression levels of *AaCht*5-1, *AaCht*5-2, *AaIDGF*2 and *AaIDGF*4 were detected in integuments; *AaCht*1-2, *AaCht*-New 1, *AaCht*-New 2 and *AaCht*-New 3 were highly expressed in the midgut, whereas all genes showed relatively low expression in malpighian tubes (Fig. 5).

### Verification of effective of RNAi

**Fig. 6:**
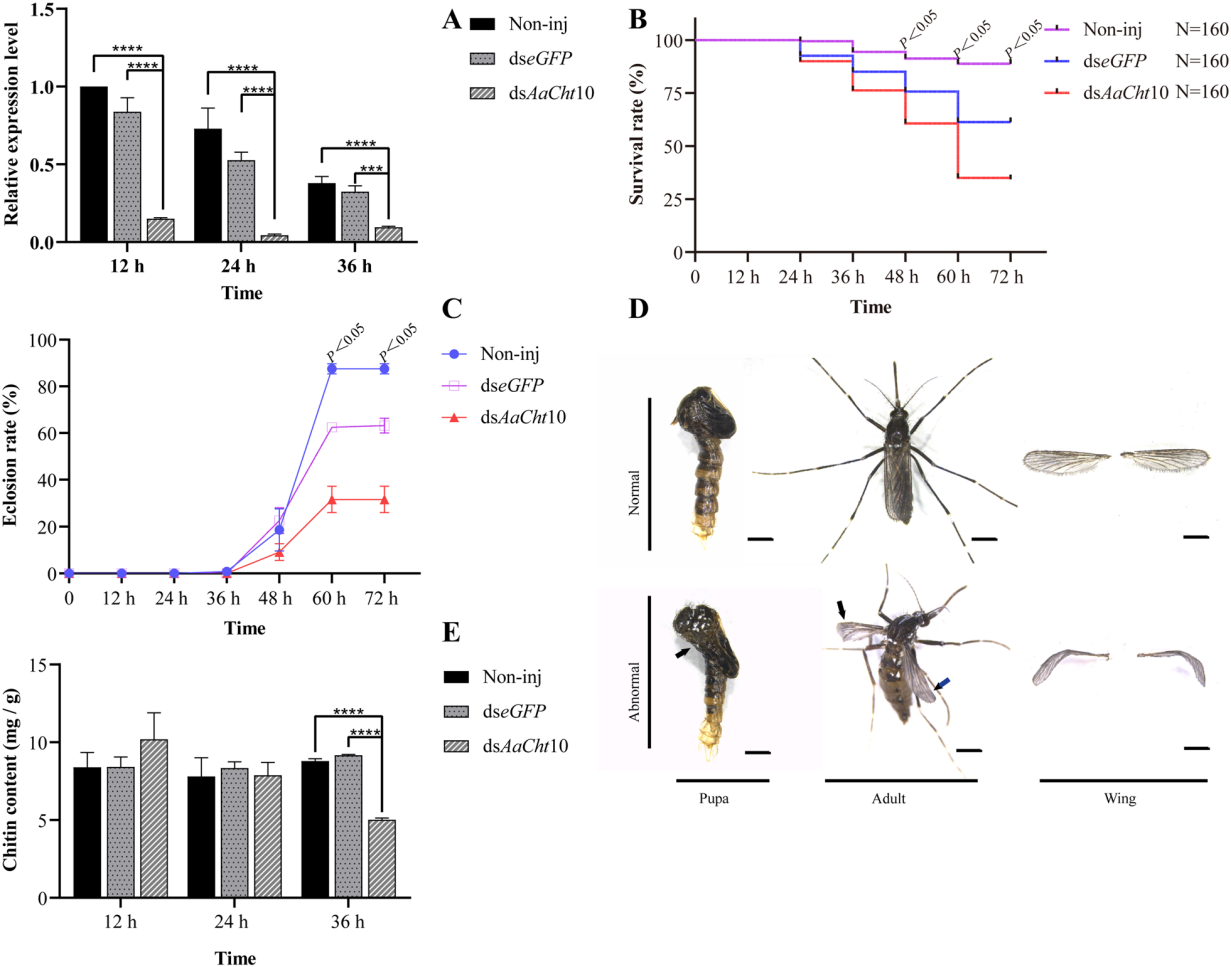
Effects of knockdown of *AaCht*10. **A** Effectiveness of RNAi; **B** survival rate of pupae; **C** eclosion rate of pupae; **D** malformed phenotype of pupae and aduls. Scale bar = 1 mm. **E** Chitin content in pupae. Non-inj, blank control; ds*eGFP*, negative control; ds*AaCht*10, treatment. ***P < 0.001, ****P < 0.0001

After injection of dsRNA, qRT-PCR was performed to evaluate gene expression levels at 12 h, 24 h and 36 h, respectively. Results showed that the expression levels of *AaCht*10 were significantly downregulated by 84.9% (*P* < 0.0001), 93.8% (*P* < 0.0001) and 74.7% (*P* < 0.0001) compared with the Non-inj group and suppressed by 82.1% (*P* < 0.0001), 92.0% (*P* < 0.0001) and 69.0% (*P* = 0.0003) compared with the ds*eGFP* group (Fig. 6A).

### Effects of deficiency of *AaCht10* on pupae

One hundred sixty pupae in each group were used to evaluate the survival rate, and results suggested that, compared with the Non-inj group, the mortality of pupae was significantly increased at 48 h, 60 h and 72 h after injection of ds*AaCht*10. At 72 h, the survival rate of the treated group was reduced to 34.4%, which was significantly decreased by 61.3% (*P* < 0.05) and 43.9% (*P* < 0.05) compared to the Non-inj group and ds*eGFP* group, respectively (Fig. 6B; Table 2). Correspondingly, a significant difference in eclosion rate was detected at 60 h and 72 h. Only 55 pupae in the ds*AaCht*10-treated group were in eclosion, while 98 and 142 pupae were successfully in eclosion in the ds*eGFP* group and Non-inj group (Fig. 6C; Table 3). Two kinds of malformations were detected in adult mosquitoes in the ds*AaCht*10-treated group. The first was splitting of the pupal cuticle, where adult mosquitoes failed to detach from the pupal shell completely; in the second, newly emerged adult mosquitoes could not fly because of deformed wings (Fig. 6D). The malformation rate in the ds*AaCht*10-treated group was up to 15%.

**Table 2:** Statistics of survival rate of pupae after RNAi of* AaCht10*

	24 h	36 h	48 h	60 h	72 h
Non-inj (% ± SE)	159/160 (99.4% ± 0.9)	151/160 (94.4% ± 1.1)	146/160 (91.2% ± 1.0)	142/160 (88.8% ± 0.8)	142/160 (88.8% ± 0.8)
ds*eGFP* (% ± SE)	148/160 (92.5% ± 2.2)	136/160 (85.0% ± 2.2)	121/160 (75.6% ± 2.4)	98/160 (61.3% ± 1.1)	98/160 (61.3% ± 1.1)
ds*AaCht*10 (% ± SE)	144/160 (90.0% ± 2.2)	122/160 (76.3% ± 2.2)	97/160 (60.6% ± 2.7)	56/160 (35.0% ± 1.3)	55 /160 (34.4% ± 1.3)

x/y: x represents the number of survivors; y represents the total number of pupae used in this experiment

**Table 3:** Statistics of eclosion rate of pupae after RNAi of* AaCht10*

	24 h	36 h	48 h	60 h	72 h
Non-inj (% ± SE)	0	1/160 (0.6% ± 0.2)	37/160 (23.1% ± 2.0)	142/160 (88.8% ± 0.75)	142/160 (88.8% ± 0.75)
ds*eGFP* (% ± SE)	0	0	33/160 (20.6% ± 1.0)	97/160 (60.6% ± 0.96)	98/160 (61.3% ± 1.1)
ds*AaCht*10 (% ± SE)	0	0	17/160 (10.6% ± 0.7)	55/160 (34.4% ± 1.3)	55/160 (34.4% ± 1.3)

x/y: x represents the number of eclosion; y represents the total number of pupae used in this experiment

**Fig. 7:**
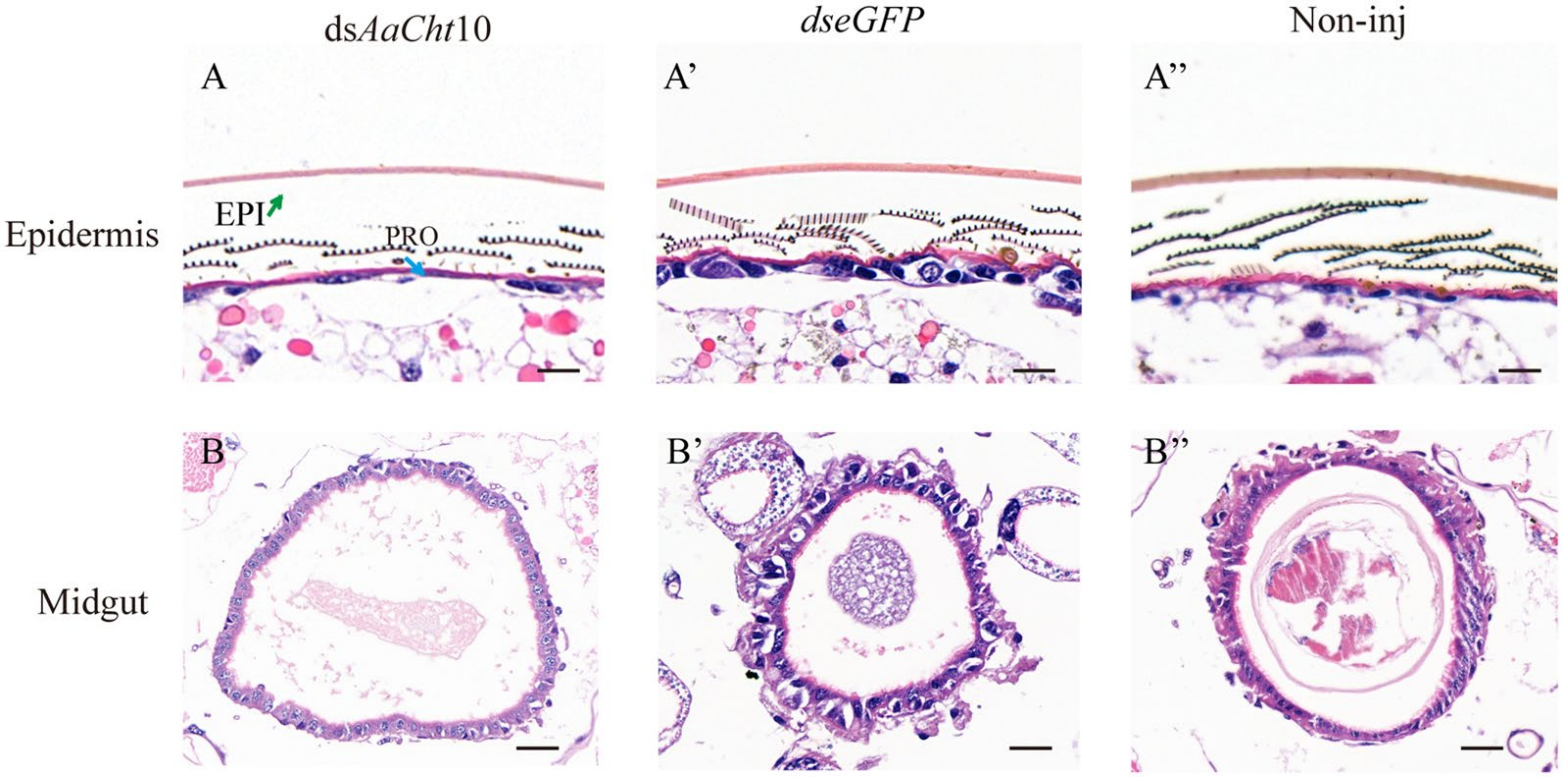
Microstructure of epidermis (**A-A’’**) and midgut (**B-B’’**) under hematoxylin and eosin staining. EPI, epicuticle; PRO, procuticle. Scale bar = 20 μm. ds*AaCht*10, treatment; ds*eGFP*, negative control; Non-inj, blank control

The chitin content assay indicated that there was no significant difference in the chitin content among groups at 12 h and 24 h after silencing of *AaCht*10. However, the average chitin content of pupae in the ds*AaCht*10-treated group was 5.0 mg/g at 36 h, much lower than that of ds*eGFP* (9.2 mg/g) (*P* < 0.0001) and Non-inj groups (8.8 mg/g) (*P* < 0.0001) (Fig. 6E). The result of H&E staining suggested that the average thickness of the epicuticle in the ds*AaCht*10-treated group was 1.49 ± 0.1 µm, which was obviously thinner than in the ds*eGFP* (2.42 ± 0.14 µm) and Non-inj groups (2.56 ± 0.06 µm); the thickness of the procuticle (1.05 ± 0.03 µm) in the treated group showed no significant difference compared with the ds*eGFP* group (1.35 ± 0.15 µm), but it was much thinner than in the control group (2.23 ± 0.24 µm) (Fig. 7A). A similar result was also detected in the midgut; the midgut wall in the treated group (12.17 ± 0.21 µm) was significantly thinner than in the ds*eGFP* (18.89 ± 0.35 µm) and Non-inj (16.14 ± 0.19 µm) groups (Fig. 7B).

## Discussion

Chitin represents up to 60% of dry weight in some insect species, which illustrates the importance of this component for insect survival [41]. As one of the structural components essential for insect growth and development, many studies have attempted to disrupt the regulatory pathways of chitin biosynthesis and degradation to control pests [42–45].

Chitinases are a large family of enzymes that degrade chitin by hydrolysis [46]; they differ substantially in their enzymatic properties, stage- and tissue-specific expression, domain organization and size [30, 31, 47]. A total of 17 Cht-related proteins were identified based on a genome-wide screen of the *Ae*. *albopictus* genome in this study. Previous research demonstrated that insect chitinases clustered into eight groups based on phylogenetic analysis of their catalytic domains [48], while *AaCht*s were clustered into seven groups. Except for several members that were in accordance with the previous model of evolution of the chitinase family GH18 [25, 48], most of the *AaCht*s were gathered with chitinases of *Ae. aegypti* and *Cx. quinquefasciatus* in group IX (Fig. 3). Cht1 and Cht3 in *D. melanogaster* were designated as portions of *DmCht*10 [15], whereas Cht1 of *Ae*. *albopictus*, *Ae. aegypti* and *Cx. quinquefasciatus* was identified and included in group IX (Fig. 3). This differential in composition of chitinase members among species may be driven by functional differentiation with the evolution of chitinase gene families. Consistent with the previous studies that showed only one member in group I in all insects with the exception of *An*. *gambiae* and *Ae. aegypti* [48], phylogenetic analysis showed that two chitinase members (*AaCht*5-1, *AaCht*5-2) were clustered into group I in *Ae*. *albopictus* (Fig. 3). Similarly, there were two members in *AaCht*1 (*AaCht*1-1, *AaCht*1-2) and *AaCht*2 (*AaCht*2-1, *AaCht*2-2), which were classified into group VII and IX, respectively. These results were caused by gene duplications resulting in two or more additional members [48, 49]. Duplicated genes may gain new structures and functions over the process of biological evolution, resulting in chitinases with different numbers among species, diverse functions and expression patterns [50, 51]. Notably, the numbers in the nomenclature of chitinases were assigned based on their sequence similarities to the correspondingly numbered Chts from other insect species. However, no orthologous genes were currently found for chitinase genes of *AaCht*-New 1, *AaCht*-New 2 and *AaCht*-New 3. Further investigations are needed to rename these chitinases and exploit their properties and physiological functions.

Domain architecture analysis demonstrated that the number of catalytic and chitin-binding domains was different among different chitinases; only *AaCht*5-1, *AaCht*10 and *AaCht*18 possess both of these two important domains (Fig. 1B). Chitin-binding domain is supposed to anchor the enzyme tightly onto the large insoluble polymeric substrate, facilitating the hydrolytic process catalyzed by catalytic domain [52, 53]. The degradative process of chitin is a dynamic process that requires coordinated action of both domains [52]. Therefore, *AaCht*5-1, *AaCht*10 and *AaCht*18 would be priority selections as a target used for *Ae*. *albopictus* control. According to previous studies, the glutamate residue (E) in motif II is the most critical residue, which is likely to be the proton donor required for cleavage of the glycosidic bond. Replacement of this residue with others resulted in total loss of activity [54]. Except for *AaCht*2-1, *AaCht*2-2, *AaCht*5-1a, *AaCht*7 (a, b), *AaCht*10 (b, c, d) and *AaCht*11, residue E in the amino acid sequence of all other chitinases has been replaced by other residues (Fig. 2), indicating that catalytic ability of these chitinases might be inactive. In addition, the developmental and tissue expression patterns suggested that chitinase genes are stage- and tissue-specific (Figs. 4, 5), which further support that these genes may have distinct functions involved in the specific stage transition and turnover of chitin in specific tissue. Results of these analyses will give clues toward choosing a suitable chitinase as a candidate target for mosquito control.

Although this is the first experimental evidence for the potential function of chitinase in *Ae*. *albopictus* to our knowledge, several studies have demonstrated that chitinases (e.g. Cht10) are essential for insect survival, molting and development [19–22, 29, 33, 49, 55, 56]. Based on the results of this study, *AaCht*10 contains both catalytic and chitin-binding domains, retains residue E in motif II and is expressed in multiple stages and tissues. The function of *AaCht*10 was verified using RNAi, and results suggested that injection of ds*AaCht*10 caused high mortality (about 65.6% in the injected group) (Fig. 6B; Table 2) and significantly reduced the eclosion rate of pupae (Fig. 6C; Table 3). Furthermore, several adult survivors after dysfunction of *AaCht*10 displayed defective morphology (e.g. failed to shed the old cuticle, wrinkled wings) (Fig. 6D). Similar results were also reported in *P. xylostella* and *Sogatella furcifera*; silencing of *PxCht*10 and *SfCht*10 caused high mortalities and lethal phenotypes [57, 58]. The high levels of mortality and developmental arrest after silencing *AaCht*10 demonstrated that *AaCht*10 is crucial for shedding of the old cuticular shell and eclosion of pupae, which can be used as a promising target to disrupt the pupa-adult transition and develop an efficient pesticide for the control of* Ae*. *albopictus*.

In addition, the suppression of *AaCht*10 had a negative impact on chitin content of *Ae*. *albopictus* (Fig. 6E), and this result was corroborated by the microstructure shown by H&E stain, which showed that RNAi of *AaCht*10 resulted in a thinner epicuticle, procuticle and midgut wall (Fig. 7). However, the previous research on *D. melanogaster* suggested that chitin content was significantly increased in wings of Cht10 knockdown flies [33]. Given that enzymatic properties and tissue-specific expression of chitinases vary with species [30, 31, 47], the inconformity results after dysfunction of Cht10 may be caused by the different tissues used to observe microstructure and quantify chitin content. The significantly reduced thickness of epicuticle, procuticle and midgut wall (Fig. 7) may be caused by reduction of chitin synthesis after dysfunction of *AaCht*10. Nonetheless, the mechanisms by which *AaCht*10 influences cuticle metabolism need to be explore in future studies.

## Conclusion

In summary, we performed a systematic genome-wide analysis of chitinase genes in *Ae*. *albopictus*. Structural features and expression patterns of 17 candidate chitinases were generated, which provide the first comprehensive information for chitinase genes of *Ae*. *albopictus* to our knowledge. Differences in their biochemical properties reinforce the notion of distinctive biological functions for specific *AaCht*. The high efficiency of *AaCht*10 in disrupted pupa-adult transition presents an opportunity to make use of *AaCht*s as efficient targets in mosquito control.

## Supplementary Information

**Additional file 1: Table S1.** Information on insect protein sequences used for phylogenetic analysis. The protein symbols of *Aedes albopictus* and *Ae. aegypti* are temporary names used in this study. **Table S2.** Primers used for qRT-PCR. **Table S3.** Primers of ds*AaCht*10 and ds*eGFP*.

## Data Availability

The authors declare that all the data related to this study are cited in the text, and data are also available in additional files.
